# Community-acquired *Klebsiella pneumoniae *meningitis in an alcoholic patient with an infected pancreatic pseudocyst; a case report and review of literature

**DOI:** 10.1186/1752-1947-1-116

**Published:** 2007-10-29

**Authors:** Adriana Orzechowska, Sandra Lacey, Geraldine Soosay, Mark Melzer

**Affiliations:** 1 Department of Microbiology, King George Hospital, Goodmayes, Essex, UK

## Abstract

We report a case of a 49-year-old male with a history of chronic alcoholism and evidence of a pancreatic pseudocyst on CT scanning. He presented with a 3-days history of fever, loss of appetite and upper abdominal pain. Blood cultures grew *Klebsiella pneumoniae *and he improved clinically with a seven-day course of intravenous co-amoxiclav and metronidazole. Two weeks later he was readmitted to hospital with impaired consciousness and septic shock, and died three days later in intensive care. Post mortem examination revealed bacterial meningitis and an infected pancreatic pseudocyst. *Klebsiella pneumoniae *was isolated from the pancreas and meninges.

## Case report

A 49-year-old man was admitted to hospital with a three day history of fever, loss of appetite and upper abdominal pain. He was of Indian origin, lived in the UK and denied any recent travel. Fifteen years previously, he was diagnosed with alcohol-related chronic pancreatitis. On admission his temperature was 38.5°C, pulse rate 142/minute, respiratory rate 24/minute and blood pressure 116/80 mmHg. He was conscious and orientated and had no meningism. Abdominal examination revealed tenderness in the right hypochondrium. White blood count was 8.5 × 10^9^/l, haemoglobin 11.5 g/dl and platelet count 200 × 10^9^/l. His CRP was 38.5 mg/L, albumin 32 g/L, bilirubin 16 umol/L, alanine-aminotransferase (ALT) 24IU/L and amylase 48 IU/L. Urinalysis revealed no nitrites or leucocytes. Blood cultures grew *Klebsiella pneumoniae*, resistant to ampicillin but susceptible to amoxicillin/clavulanic acid by BSAC disc diffusion methods. Abdominal CT scan with contrast revealed evidence of a pseudocyst in the pancreas but no evidence of infection. Radiologically, there was no evidence of cholelithiasis, obstruction of the common bile duct or cholecystitis. Intravenous (iv) antibiotics, amoxicillin/clavulanic acid 1.2 g, thrice daily, (tds) and metronidazole 500 mg tds, were commenced with one dose of gentamicin 400 mg (iv). Antibiotics were continued for seven days. The patient improved clinically and was discharged home. The source of his bacteraemia remained undefined.

Two weeks later the patient was readmitted with a four day history of rigors. On admission, his temperature was 40°C, O_2 _saturation 98%, pulse 142/min and BP 140/90. White cell count was 10.6 × 10^9^/l, haemoglobin 10.6 g/dl and platelet count 199 × 10^9^/l. His CRP was elevated at 236.1 mg/L. Liver function tests were normal. Blood culture again grew *Klebsiella pneumoniae*, resistant to ampicillin. Prior to blood culture results, empirical treatment with piperacillin/tazobactam (iv) 4.5 g tds was commenced. Four hours later, the patient became unresponsive and his Glascow Coma Score (GCS) fell to 6/15. An arterial blood gas revealed metabolic acidosis with respiratory compensation. CT scan of the head was normal. The patient was transferred to ITU, intubated, ventilated and maintained on the same antibiotic regime. Two days later he died. A post mortem revealed an infected pancreatic pseudocyst and bacterial meningitis. Tissue and pus from both sites grew *Klebsiella pneumoniae*. Pancreatic, blood and meningeal *K pneumoniae *isolates were sent for typing to the UK Health Protection Agency (HPA) Enteric Pathogen laboratory. All isolates had indistinguishable serotypes (K2) and a pulse field gel electrophoresis pattern designated 'King 15'.

## Discussion

Like other members of the family enterobacteriaceae, *K pneumoniae *causes infection of bile and urine, sterile fluids in close proximity with the gastrointestinal tract. Complications of primary sites of infection are uncommon but liver and prostatic abscesses do occur. Dissemination of *K. pneumoniae *to secondary sites is rare but septic arthritis, vertebral osteomyelitis and endophthalmitis have been described.

In adults, *S. pneumoniae *and *N. meningitides *are the commonest causes of bacterial meningitis. Bacterial meningitis caused by *K. pneumoniae *is uncommon but some cases have been reported, especially in Taiwan. Over a four-year period, 27 Taiwanese patients were diagnosed with *K. pneumoniae *meningitis[1] and in another Taiwanese case series conducted over six years, the proportion of *K. pneumoniae *as a cause of bacterial meningitis rose from 8% to 18%[2]. Underlying co-morbidities included diabetes, alcoholism and chronic liver disease[1-4]. Outside Taiwan, cases of *K. pneumoniae *meningitis have occurred, predominantly in other parts of Asia [5-7] but also in Europe [8-11] and North America [12-14]. The rarity of these cases outside Asia raises the possibility of ethnicity or country of origin predisposing individuals to invasive disease[15].

Phenotypically, *K. pneumoniae *isolates with hypermucoviscosity are, 'in-vivo', anti-opsonic, anti-phagocytic and associated with severe disease[16]. Isolates with K1 and K2 capsular antigens are the most invasive pathogens. These capsular serotypes, rather than the genes *magA *and *rmpA *that regulate extracapsular polysaccharide synthesis, are probably the most important virulence determinants for *K. pneumoniae*[17].

Clinical outcomes are poor in cases of *K pneumoniae *meningitis despite adequate and prolonged antibiotic treatment. Different choices of empirical intravenous cephalosporins do not alter clinical outcomes[18]. In the fore-mentioned case series, other prognostic factors of patients were analysed. Time from onset of symptoms to start of appropriate therapy, disease severity, age, gender, diabetes mellitus, acquisition settings and antibiotic resistance were not significantly correlated with survival. The only determinant of a better neurological and clinical outcome was the timing of the first dose of appropriate antibiotic given before levels of consciousness fell below a GCS of eight.

Pancreatic pseudocysts are an uncommon complication of pancreatitis. They arise from disruptions of the pancreatic duct due to pancreatitis and extravasation of enzymatic material. Over 75% of cases are caused by alcohol and most pseudocysts resolve spontaneously. The optimal management of pancreatic pseudocysts remains debatable although drainage should be performed when symptoms persist or complications arise. Infection occurs in approximately 10% of cases[19] and this is an indication for drainage which can be achieved endoscopically or at open surgery.

## Conclusion

To our knowledge this is the first case of an infected pancreatic pseudocyst to cause *K. pneumoniae *bacteraemia. Remarkably, it caused bacterial meningitis following initial clinical improvement with a seven-day course of appropriate antibiotics. Had we suspected the pseudocyst was infected we would have advised drainage and a longer course of treatment. Rarely, outside Asia, and in alcoholic patients with underlying co-morbidities, virulent strains of *K. pneumoniae *may disseminate from primary sites of infection and cause bacterial meningitis.

## Competing interests

The author(s) declare that they have no competing interests.

## Authors' contributions

AO wrote the case history and researched the discussion. MM and SL were involved in the patient's management and gave antimicrobial advice. GS performed the post mortem. All authors have read and approved the final manuscript.

## Consent

Written informed consent was obtained from the patient's relative for publication of this case report.
